# Empirical Determination and Modelling of Compressive Strength of Cement Composites with Waste Tyre Rubber

**DOI:** 10.3390/ma19183993

**Published:** 2026-09-19

**Authors:** Todorka Samardzioska, Silvana Petruseva, Milica Jovanoska-Mitrevska, Slobodan B. Mickovski, Vladimir Vitanov

**Affiliations:** 1 Faculty of Civil Engineering, Ss. Cyril and Methodius University in Skopje, 1000 Skopje, North Macedonia; silvana@gf.ukim.edu.mk (S.P.); m.jovanoska@gf.ukim.edu.mk (M.J.-M.); slobodan.mickovski@gcu.ac.uk (S.B.M.); v.vitanov@gf.ukim.edu.mk (V.V.); 2 Department of Construction and Built Environment, Glasgow Caledonian University, Glasgow G4 0BA, UK

**Keywords:** waste tyre rubber, cement composites, compressive strength prediction, machine learning, support vector machine, artificial neural networks

## Abstract

Incorporating waste tyre rubber into cement composites could pose a sustainable strategy for reducing tyre waste, whilst simultaneously preserving natural resources. Accurate prediction of the compressive strength is essential for the efficient development and application of the new material. This study presents the experimental research and a new proposed machine learning modelling framework aimed at predicting the compressive strength of conventional and rubber-modified cement composites containing recycled rubber derived from end-of-life vehicle tyres. Experimental datasets from compressive strength tests on 48 concrete and 40 mortar specimens were used to develop predictive models, with varying rubber content. The empirical results show that increasing the percentage of waste rubber in the mixture designs negatively affects compressive strength, reducing it up to 96.06% for mortars, up to 64.4% for concrete made with ordinary Portland cement, and up to 60.7% for concrete made with sulfate-resistant cement. Three soft computing techniques were implemented using the DTREG predictive modelling software: the General Regression Neural Network (GRNN), Radial Basis Function Neural Network (RBFNN), and Support Vector Machine (SVM). The conventional linear regression (LR) model was also implemented and compared with these three machine learning models. They were trained using the experimentally measured results for the various mixtures. The model performance was evaluated using the standard estimators: the coefficient of determination (R^2^), correlation coefficient (r), and mean absolute percentage error (MAPE). The GRNN demonstrated the best predictive abilities for predicting the strength of mortars, with MAPE = 4.6% and R^2^ = 99.27% on the original dataset; on the normalized dataset, MAPE was 3.9%, and R^2^ was 99.65. For predicting the strength of concrete, GRNN also outperformed the other models on the original dataset with MAPE = 3.8% and R^2^ = 98.4%. The rubber content was identified as the most influential parameter affecting the compressive strength, while density, admixture conditions, the water-cement ratio, and curing age also contributed to the model’s performance. The LR model presented the lowest predictive accuracy among all evaluated algorithms (models) because of the significant complex nonlinear interactions between predictors and target, which can not be adequately modelled with a linear framework. Within the investigated experimental domain, the developed models showed high predictive accuracy and can be used for preliminary strength estimation and interpolation for mixtures with similar characteristics.

## 1. Introduction

Approximately one billion tires reach the end of their service life each year, posing a serious global environmental problem [1,2]. Although the total volume of municipal waste in Macedonia saw a slight increase (1.2%) between 2021 and 2025, waste from used vehicle tyres decreased by 30.8% in 2025 compared to the 1598 tons recorded in 2021. Nevertheless, 99.8% of all municipal waste in Macedonia is sent to landfills [3]. On the other hand, natural resources and fossil energy sources are increasingly being consumed and depleted, impacting the environment and greenhouse gas emission levels.

The construction sector has recently embraced the challenge of integrating sustainability into production processes by identifying more eco-friendly raw materials, incorporating solid waste into concrete as aggregates, and studying its properties. The use of waste rubber in sustainable building materials is one of the several solutions for reducing rubber waste and protecting the environment. The use of waste rubber as an aggregate in cement-based building materials has been investigated in many studies; however, its use in asphalt materials is assessed as more attractive [4,5]. Many researchers tested the physical and mechanical properties of the fresh mixtures and hardened cement materials. The interaction between the rubber and the cement matrix was studied, as well as the workability, consistency, porosity, and density of fresh mortars and concretes. The influence of rubber participation on the properties of the hardened mortars and concretes was observed [5]. Mechanical and rheological properties, such as compressive and flexural strength, insulation ability (thermal and acoustical), wettability, and water impermeability, are of interest to scientists in this field of research.

Hasan et al. [6], Jusoh et al. [7], Moreno et al. [8], Senin et al. [9], and Yedra et al. [10] are just some of the authors of papers in the field of research on the properties of mortars with the addition of waste rubber. Various properties of the mortar mixtures and hardened mortar have been analysed, and different conclusions from the research have been published.

Due to their outstanding strength, flexibility, and stress control attributes, rubber granules are commonly used in cement composites as a replacement for fine (≤4.75 mm) and coarse aggregates (≤20 mm). For untreated rubber that has not undergone any solvent treatment, it can be observed that its addition to concrete can improve its flowability [11]. According to previous research, the addition of 15% rubber granules can increase the workability from 50 mm to 75 mm [12]. Similarly, published research shows that the inclusion of rubber effectively improves the workability of concrete.

Due to the poor bond between the cement matrix and the rubber, many researchers choose to treat the rubber particles with 10% NaOH before incorporating them into the concrete, which helps to improve the interaction between the concrete and the rubber particles. The authors Mohammadi et al. [13] and Moases et al. [14] showed that NaOH-treated rubber concrete exhibits superior mechanical properties compared to untreated rubber concrete.

Evidence suggests that replacing fine aggregate with rubber from waste tyres at a proportion of up to 3.5% does not significantly affect compressive strength [1]. Adding a larger amount of rubber reduces mechanical properties. However, the use of rubber increases the concrete’s ductility, improves durability, and reduces its density, making it suitable for lightweight concrete [1,2].

The optimal ratio of sulphur quantity, rubber content, and micro-filler in the sulphur concrete mixture—with added rubber—has been investigated in terms of compressive strength by [15]. Due to its rapid hardening and early strength development, this material can be used to manufacture prefabricated products such as bricks, noise barriers, and lightweight wall panels, as well as for 3D printing.

A mixture containing 10% rubber (by mass) has been tested [16] to evaluate the compressive strength of concrete incorporating recycled rubber as an aggregate for structural applications requiring a minimum compressive strength of 25 MPa, aiming to maintain workability, flowability, and structural load-bearing capacity. This rubberized concrete exhibited a reduction in compressive strength of only 2 MPa compared to conventional concrete. A density reduction of 4.31% was achieved, making this material a competitive and environmentally sustainable alternative to conventional concrete, particularly for applications where weight reduction is a priority.

The type and size of rubber particles can significantly influence the characteristics of cementitious materials. Fine rubber particles have been used as a substitute for sand, and shredded rubber as a substitute for stone aggregate, at replacement levels of 5%, 10%, and 15% by weight [17]. The results showed that increasing the rubber content improved Poisson’s ratio, ductility, workability, and toughness significantly compared to conventional concrete, despite the reduced density, compressive strength, and tensile strength. Furthermore, AlMousawi et al. [18] used three types of rubber particles: rubber powder, rubber granules, and rubber chips, with replacement rates of 10% and 20% by volume. Compressive strength was evaluated at 7, 14, and 28 days, and showed that increasing the rubber content and particle size led to a reduction in workability and compressive strength. Large rubber particles (chips) caused the formation of voids and micro-cracks within the matrix, indicating poor adhesion in the interfacial transition zone. In contrast, Afannoue et al. [19], using three types of rubber particles, demonstrated that the coarser the particles, the better the strength retention. Specifically, at a 20% replacement level, compressive strength decreased by 24% for rubber powder, 20% for rubber sand, and 17% when using coarse rubber aggregates.

Waste rubber has also been investigated for use in high-strength concrete [20]. Microscopic morphological analysis indicates that reducing the size (volume and specific surface area) of rubber particles strengthens their bond with the matrix. This study demonstrates that high-quality rubberized concrete has the potential to improve the durability and sustainability of construction projects. Structures built with this type of concrete can withstand greater loads and impact forces.

Despite extensive experimental work, the prediction of the compressive strength of this type of concrete remains insufficiently developed. The study by Sinkhonde et al. [21] proposes, for the first time, a method for predicting the compressive strength of rubberized concrete using models based on artificial neural networks (ANN). The prediction relies on multilayer perceptron (MLP) networks and radial basis function (RBF) neural networks, with the MLP model demonstrating superior performance. For both methods, R^2^ values exceed 0.75.

Based on a literature review, Hadzima-Nyarko et al. [22] assert that few studies have been conducted using ANNs to predict the compressive strength of rubberized concrete, and that no algorithm exists for determining the compressive strength of such concrete. They compiled a large database containing 457 experimental results from the literature, which is necessary to obtain a more reliable estimate of compressive strength. The model with three hidden layers gave the best results with respect to prediction accuracy-highest R values of 0.96 and 0.98 for the train and test data, respectively—and lowest RMSE (4.8 for the train and 3.78 for the test data) and MAPE with respective values of 20.2 for the train data and 21.6 for the test data.

Furthermore, the study by Chong et al. [23] utilized 33 datasets regarding concrete with waste tire rubber as a partial replacement for fine aggregates. Their model, based on the Response Surface Methodology (RSM) for predicting the compressive strength of rubberized concrete, demonstrated high accuracy (R^2^ = 0.9923, RMSE = 2.368). The Regression analysis of the strength index for rubberized concrete indicated that the loss of strength in this type of concrete can be expressed through an exponential function of the replacement percentage.

To improve the accuracy of predicting the strength of rubberized concrete, a study by Huang et al. [24] developed an artificial neural network model utilizing a hybrid optimization algorithm, which enhanced the network’s performance and accuracy. The results demonstrated that the hybrid algorithm can be used to optimize neural networks for predicting the strength of rubberized concrete and holds potential for further improvement.

Dat et al. [25] also proved that the ANN model is more reliable and stable than the other models and can be considered a suitable approach for predicting the compressive strength of eco-friendly concrete containing waste tire rubber. They compared an artificial neural network (ANN) model with three hidden layers with two machine learning techniques—Random Forest and Multilayer Perceptron—for predicting the compressive strength of concrete containing waste tire rubber, using a dataset of 129 samples from technical literature.

Incorporating rubber into cementitious materials increases flexibility and elasticity, thereby improving crack resistance Vadivela et al. [26]. Their study explores the potential use of rubberized concrete in applications where impact resistance is critical. The developed machine learning model showed promising results in predicting various strength properties. Model performance varies depending on the specific strength parameter being predicted and the chosen machine learning algorithm, with CatBoost demonstrating superior performance compared to other models in predicting compressive strength.

Support vector machine (SVM) and Gaussian process regression (GPR) models were used for prediction of rubbercrete compressive strength with data collected from the literature [27]. The fine aggregate was replaced with waste rubber in a range from 0% to 100% by volume. The GPR model had better results in the strength prediction.

Incorporating waste tyre rubber into cement composites could pose a sustainable strategy for reducing tyre waste while simultaneously conserving natural resources.

Several previous ML studies on rubberized concrete have used datasets compiled from published literature. In contrast, the present study uses newly generated, internally consistent experimental datasets for both rubberized mortar and concrete. The novelty lies in combining these controlled experimental datasets with a comparative evaluation of GRNN, RBFNN, SVM, and conventional LR models using mixture-grouped LOMO validation, thereby providing a modelling framework for strength estimation and interpolation within the investigated mixture space.

The aim of this research is to investigate the compressive strength of conventional and rubber-modified mortars and concretes containing recycled rubber derived from end-of-life vehicle tyres and to explore the use of predictive models for estimating this property within the investigated mixture space. Such models may support preliminary strength estimation and mixture assessment. The soft computing and regression models were developed using the DTREG predictive modelling software.

## 2. Materials and Methods

### 2.1. Materials and Mixture Designs

#### 2.1.1. Mortar Mixtures

Two types of mortar were prepared: Mortar 1 without an admixture (M1) and Mortar 2 (M2_A) with a superplasticizer admixture. Mortar M1 was produced using six different mix designs—a control mix and mixes with 10%, 15%, 20%, 30%, and 50% of the aggregate replaced by waste rubber by mass—whereas only the first four mix designs were prepared for Mortar M2_A. The detailed mix proportions for Mortars M1 and M2_A are presented in Table 1 and Table 2, respectively.

**Figure 1:**
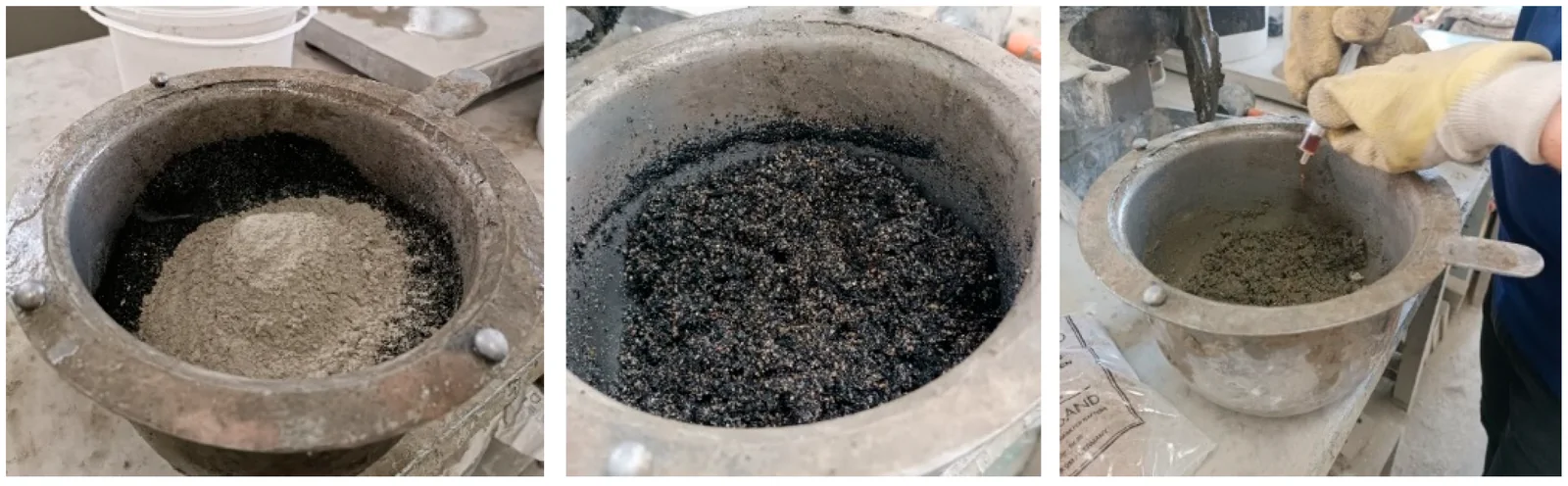
Dosage of necessary raw materials for mortar mixtures.

Portland cement (CEM I) was used for the preparation of both types of mortars. In addition, a small amount of gypsum was used to regulate the setting time. Standard CEN sand was used as the aggregate—a commercially available product composed of several different sand fractions obtained through manufacture Figure 1.

The Superfluid 21M1M admixture (“Ading”, Skopje, Macedonia) was used in the preparation of the M2_A type mortar mixtures to enable a high degree of water reduction in the concrete and the production of various types of concrete with high consistency classes.

The waste rubber used was supplied by “Uni-Royal Kompani” Ltd. (Prilep, Macedonia), as an end-of-life tyre which the end-user discards, intends to discard, or is required to discard —excluding waste and residues from the processing of used tyres that are handled in accordance with international waste management regulations. The bulk density of the waste rubber was determined to be 395 kg/m^3^, in accordance with the MKS EN 1097-3 [28] standard. The particle-size distribution of the waste rubber is provided in Supplementary Figure S1. The material was predominantly fine-grained, with 96.2% passing the 4 mm sieve and 84.1% passing the 2 mm sieve.

The mortar mixtures were prepared according to MKS EN 1015-1 [29], and the test samples were made according to MKS EN 1015-2 [30].

#### 2.1.2. Concrete Mixtures

Concrete mixtures tested in this research differed in the type of cement and the percentage of waste tyres. Two types of concrete mixes were made, with regular Portland Cement (C_PC) and sulphate-resistant cement (C_SC). From both types of concrete mixes, 4 different recipes were made: a reference mix and replacements of 15%, 30%, and 50% of the 0/4 mm fine aggregate replaced by waste rubber by volume. The detailed mix proportions for concretes C_PC and C_SC are presented in Table 3 and Table 4, respectively.

Two different types of cement were used to prepare the concrete mix designs:

- CEM II/A-LL 42.5 R: Portland cement composed of 80–94% clinker, 6–20% limestone, and 0–5% other constituents. It has a strength class of 42.5 MPa and high early strength. It was used for concrete type C_PC.
- CEM I 42.5 R-SR5: Portland cement composed of 95–100% clinker and 0–5% other constituents. It has a strength class of 42.5 MPa and high early strength. It is a sulfate-resistant cement with a low tricalcium aluminate (C_3_A) content (<5%), providing significant protection against chemical aggression. It protects the concrete against groundwater and sulfate exposure caused by seawater, wastewater, or aggressive soil. It was used for concrete type C_SC.

Two different types of aggregate were used:

- River aggregate (0/4 mm) from the Aradiko Kop processing plant (Skopje, Macedonia), with a bulk density of 1590 kg/m^3^, determined in accordance with MKS EN 1097-3 [28].
- Crushed limestone aggregate (4/8, 8/16, and 16/32 mm) from the In Mak quarry (Kumanovo, Macedonia).

The same admixture and the same waste rubber from car tires as those used for the mortar were employed in the preparation of all concrete mixtures.

### 2.2. Methods

#### 2.2.1. Experimental Methods and Standards

**Figure 2:**
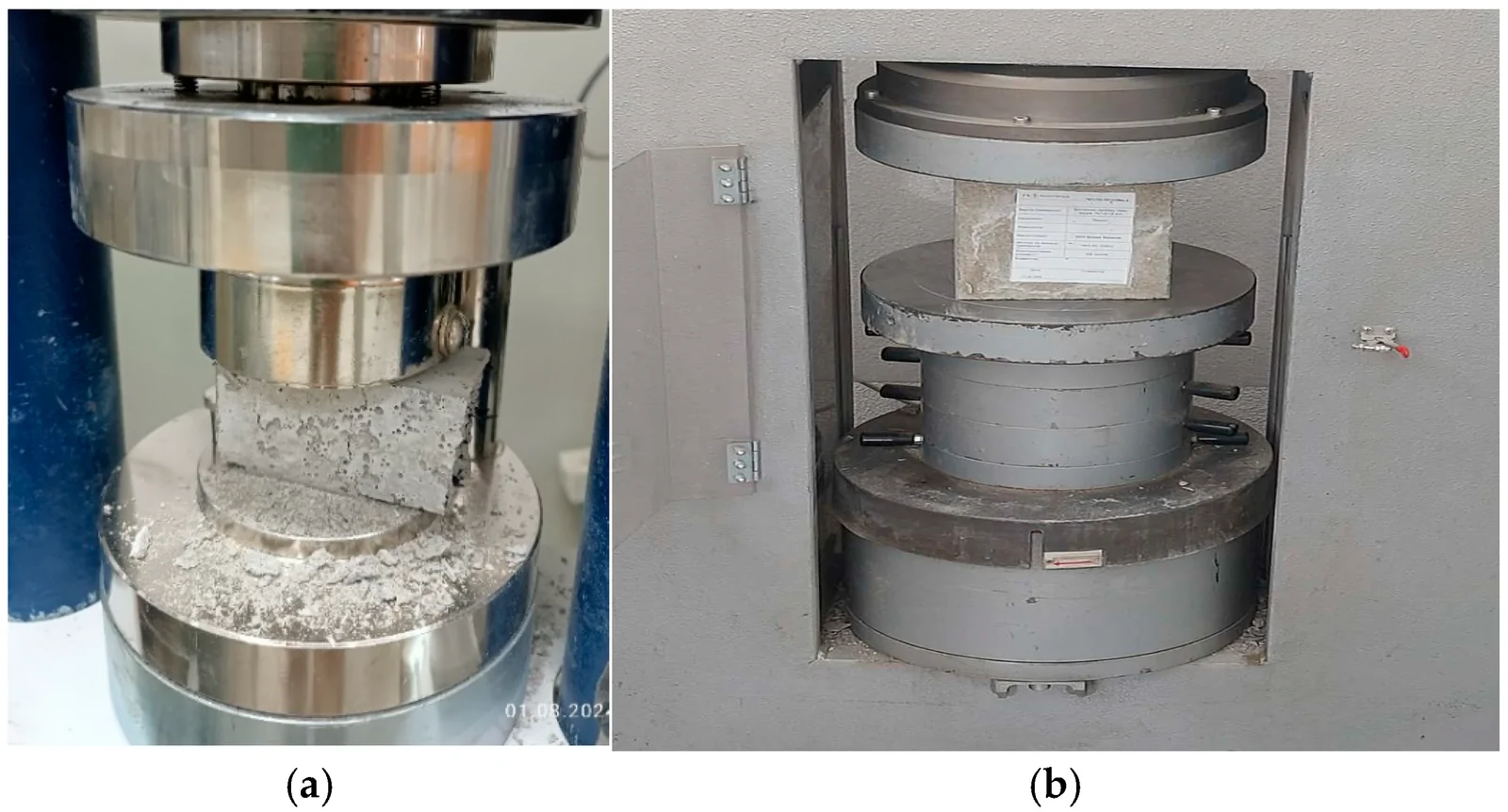
Testing of compressive strength on: (**a**) Mortar samples; (**b**) Concrete samples.

The dry bulk density of mortar samples was measured according to MKS EN 1015-10 [31]. Strength tests were conducted on hardened mortar specimens in accordance with MKS EN 1015-11 [32], as shown in Figure 2a.

The compressive strength of all concrete specimens was tested in accordance with MKS EN 12390-3 [33]. A testing machine compliant with MKS EN 12390-4 [34] was used for this purpose (Figure 2b).

#### 2.2.2. Soft Computing Methods for Prediction

The modelling for prediction of the strength of the concrete and mortar was conducted using three soft computing methods: two neural networks, the general regression neural network (GRNN) and radial basis function neural network (RBF NN), and support vector machine (SVM), using the predictive modelling software DTREG (Enterprise version 10.10.0) [35].

Soft computing methods are a set of problem-solving methods that try to implement the intelligence found in nature, and the most important and most often used of them are the methods for learning from experimental data and fuzzy logic methods. Other constituents of soft computing are probabilistic reasoning, fractals, genetic algorithms, swarm intelligence, neuro-fuzzy systems, and others [36]. In this paper, we focus on artificial neural networks (ANNs) and SVM, which implement the idea of learning from experimental data.

In the last several decades, traditional hard computing methods, which require a lot of computational time and precisely defined mathematical models, have proven inappropriate for solving today’s multidimensional nonlinear problems, especially when there is no analytical model or not enough data for solving some engineering problem. ANNs and SVMs have appeared as powerful alternatives to such traditional methods. In recent years, this technology has been used to solve many different problems in civil engineering and many other research areas because of their capability to learn only from experimental data, approximating any multivariate function and modelling highly complex nonlinear processes.

The structure of artificial neural networks is inspired by the functioning of the biological neurons in the nervous system, and their architecture contains many computing units called neurons. The connection between two neurons is called a weight, which expresses the strength of that connection. The values of the weights are subject to the learning process of the neural network (NN). There are different types of ANNs, depending on the architecture and on the amount of available data, according to the task they should solve (regression or classification task).

Neurons have at least 3 layers: input, hidden, and output layers, or there may be several hidden layers for complex tasks. Every neuron in the hidden layer has a nonlinear activation function, which adjusts the values of the weights, enabling nonlinear learning. Depending on the task being solved, the neurons in the output layer have different types of activation functions. The input layer receives the data in the form of a vector, whose components are values of the variables, predictors, and target included in the modelling, which should explain the problem mostly. The most important part of the modelling with ANNs is the selection of the most representative variables for the problem being solved.

Usually, around 70% of the available data for modelling are used for training the NN, around 10–15% are used for internal validation of the model by the algorithm of the NN, and around 15% is used for testing the accuracy of the model on unseen data. When the model demonstrates high accuracy on an unseen data set, then that NN is said to have an excellent generalization capability.

ANN will learn the relationship between the target variable which should be predicted (or classified) and predictors, using the learning algorithm of the specific NN that minimizes the error between predicted values and corresponding actual values of the target.

For developing a NN model for the behaviour of some material, the NN should be trained with a set of experimental data, which mostly explain that behaviour. If the data contains the most relevant parameters that describe the material’s behaviour, then the NN model will give sufficient information about the behaviour of that material, which can be qualified as a model of that corresponding material [37]. NN, which is trained on such data, will be able to generalize the experimental results, approximating the results on unseen data with the same variables.

An overview of the most important characteristics of the two neural networks utilized in this paper is provided in the following.

**Figure 3:**
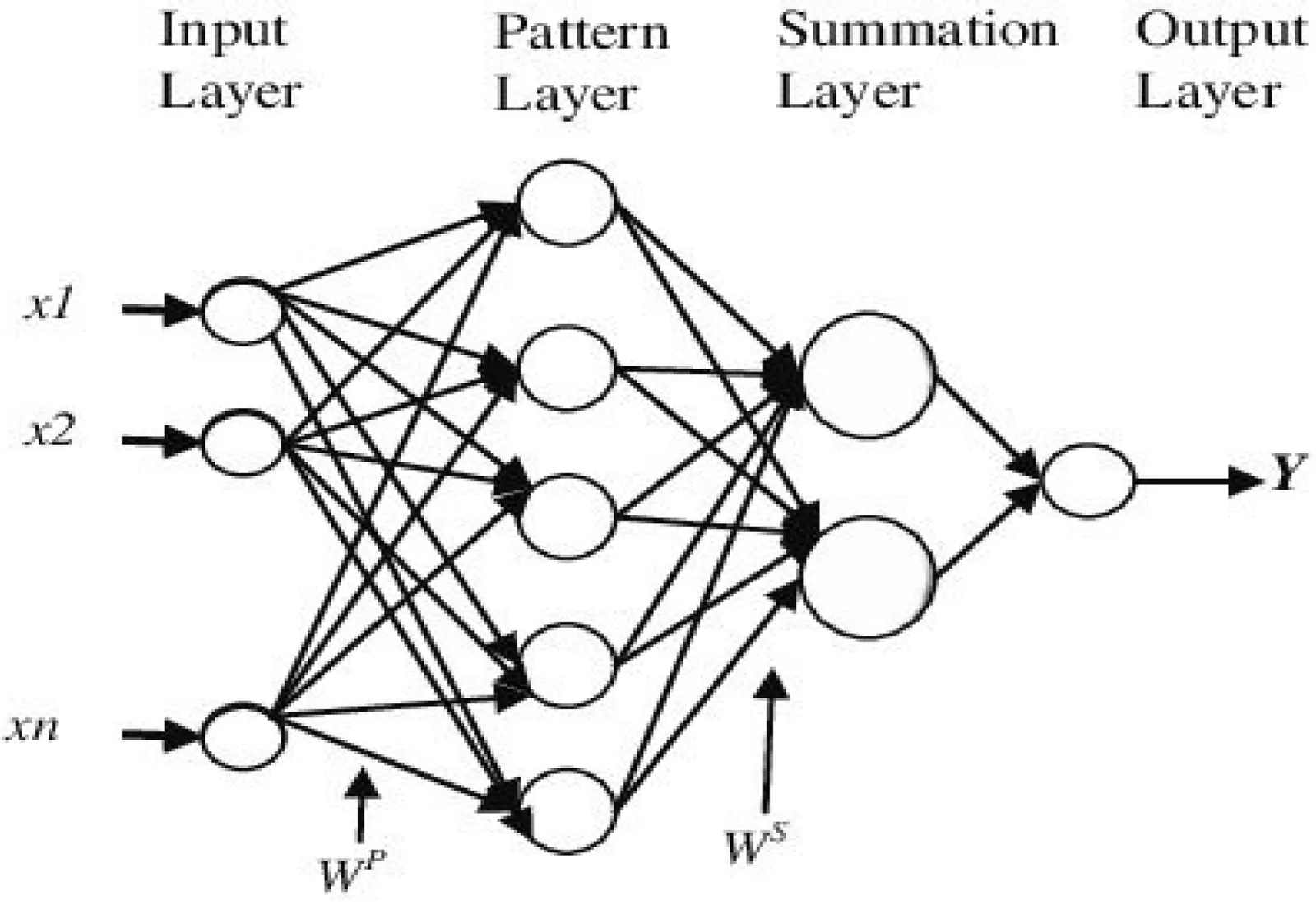
GRNN architecture.

General Regression Network (GRNN) is a neural network that is used mostly for regression problems. Specht proposed it in 1991 [38], but it has undergone several improvements till now. Because of its very attractive characteristics, some authors consider it one of the most groundbreaking neural networks in the last decades. Some of its capabilities are that it can converge to the optimum in one pass, very quickly, and the modelling is very accurate; also, it can learn from small datasets, managing noisy datasets very well. The architecture of GRNN is presented in Figure 3. One of the disadvantages of GRNN is that it is a computationally expensive NN because it needs a large amount of memory.

The four layers of GRNN are: input layer, two hidden layers pattern and summation layer and output layer. The input layer receives the vector with the values of the predictors; the number of neurons is the same as the number of predictors (*x*_1_, *x*_2_, *x*_3_, *…*, *x_n_*). The values of the input neurons are propagated to the neurons of the next pattern layer. The pattern layer has the number of neurons equal to the number of examples (rows, cases) in the dataset (which contain the values of the predictors and target values). In this layer, the Euclidean distance of every new case to the centre of the neuron (some initial value from the training set) is computed using a Gaussian function as an activation function (Equation (1)):

(1) $$G\left(x\right)=exp(-{\left\|x-x_{i}\right\|}^{2}/2\sigma^{2})$$

where ‖***x − x_i_***‖ is the distance between the new case ***x*** and the centre ***x_i_*** of the *i*-th neuron. Sigma is the smoothing parameter. The most important part of training the GRNN is adjusting the sigma values, which impact the spread of the Gaussian function.

The values from the neurons in the pattern layer are transferred to the next summation layer. This layer has 2 neurons called numerator and denominator. The numerator neuron computes the sum of weight values from the previous layer multiplied by the target values, while the denominator computes the sum of the weight values from the previous layer (from each neuron). The last output (decision) layer computes the quotient of the numerator and denominator, obtaining the predicted target value. The algorithm of GRNN implements the following formula (Equation (2)):

(2) $$\hat{Y}\left(x\right)=\frac{\sum_{i=1}^{m}y_{i}G(x)}{\sum_{i=1}^{m}G(x)}$$

where $\hat{Y}\left(x\right)$ is the predicted target value, *m* is the number of rows in the dataset, *y_i_* is the *i*-th actual target value, and *G*(*x*) is a Gaussian function (Equation (1)).

The GRNN has been used in many research areas: civil engineering, physics, medicine, and geology, and has successfully solved problems of prediction, control, classification, pattern recognition, and predictions with time series.

**Figure 4:**
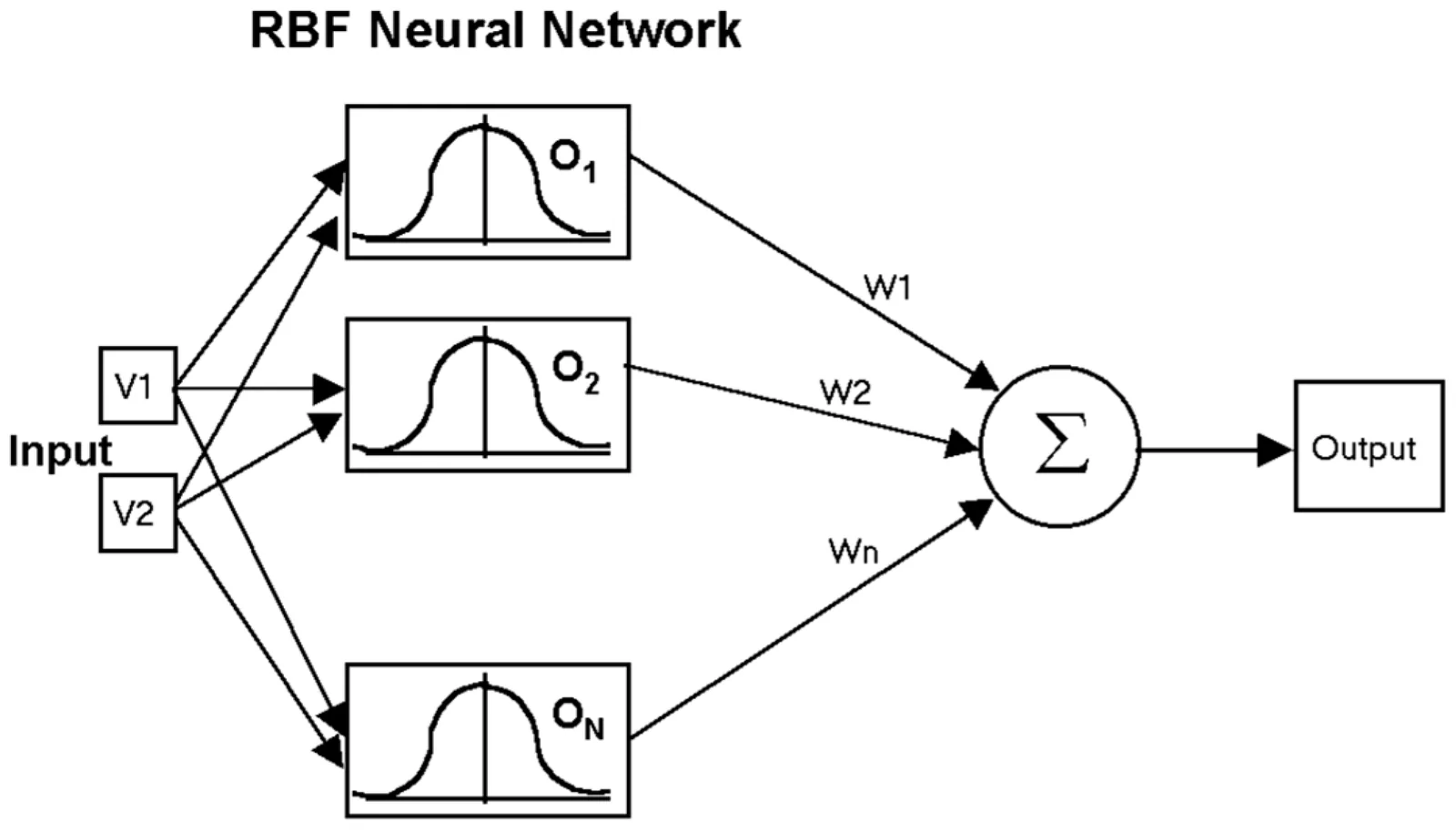
RBF NN architecture.

Radial basis function neural network (RBF NN) is a very elegant mathematical structure; it is very powerful in solving problems of classification and regression. It has only 3 layers: input, hidden, and output (Figure 4). The first mathematical foundation for RBF NN was proposed by Broomhead and Lowe [39], and in the next period several other authors have made improvements to the algorithm of this NN. In the input layer, there are the same number of neurons as the number of predictors. The values from the input layer are transferred to the neurons in the next hidden layer, which has an adjustable number of neurons, depending on the training process. The activation radial basis function of each neuron in this layer is a function of the number of variables equal to the number of predictors. When the vector ***x*** from the input layer enters this hidden layer, the Euclidean distance of vector ***x*** from the neuron’s centre is calculated, and then the RBF function is applied to this distance, and the result is propagated to the last summation layer. Every such result from the neurons in the hidden layer is multiplied by the weight value linked to that neuron and transferred to the last layer, which sums these values and yields this sum as the network’s output [35].

RBF NN implements the following formula (Equation (3)) [40]:

(3) $$y\left(x\right)=\sum_{j=1}^{M}w_{j}\phi_{j}\left(x\right)+b$$

where *y*(*x*) is the output of the network, *x* is the input vector, *M* is number of neurons in the hidden layer, *w_j_* are the weight coefficients between hidden and output layer, *ϕ_j_*(*x*) is radial basis function for the *j*-th neuron, which in the most of the cases is Gaussian function (Equation (1)), and *b* is some bias parameter. The output *y*(*x*) of the RBF NN is the linear combination of the radial basis functions in the hidden layer.

RBF NN has been applied very successfully in many areas of research: hydrology (river-flow forecasting, groundwater level prediction), time series forecasting, adaptive control systems, financial trends, signal processing (in medicine, telecommunication), and others.

**Figure 5:**
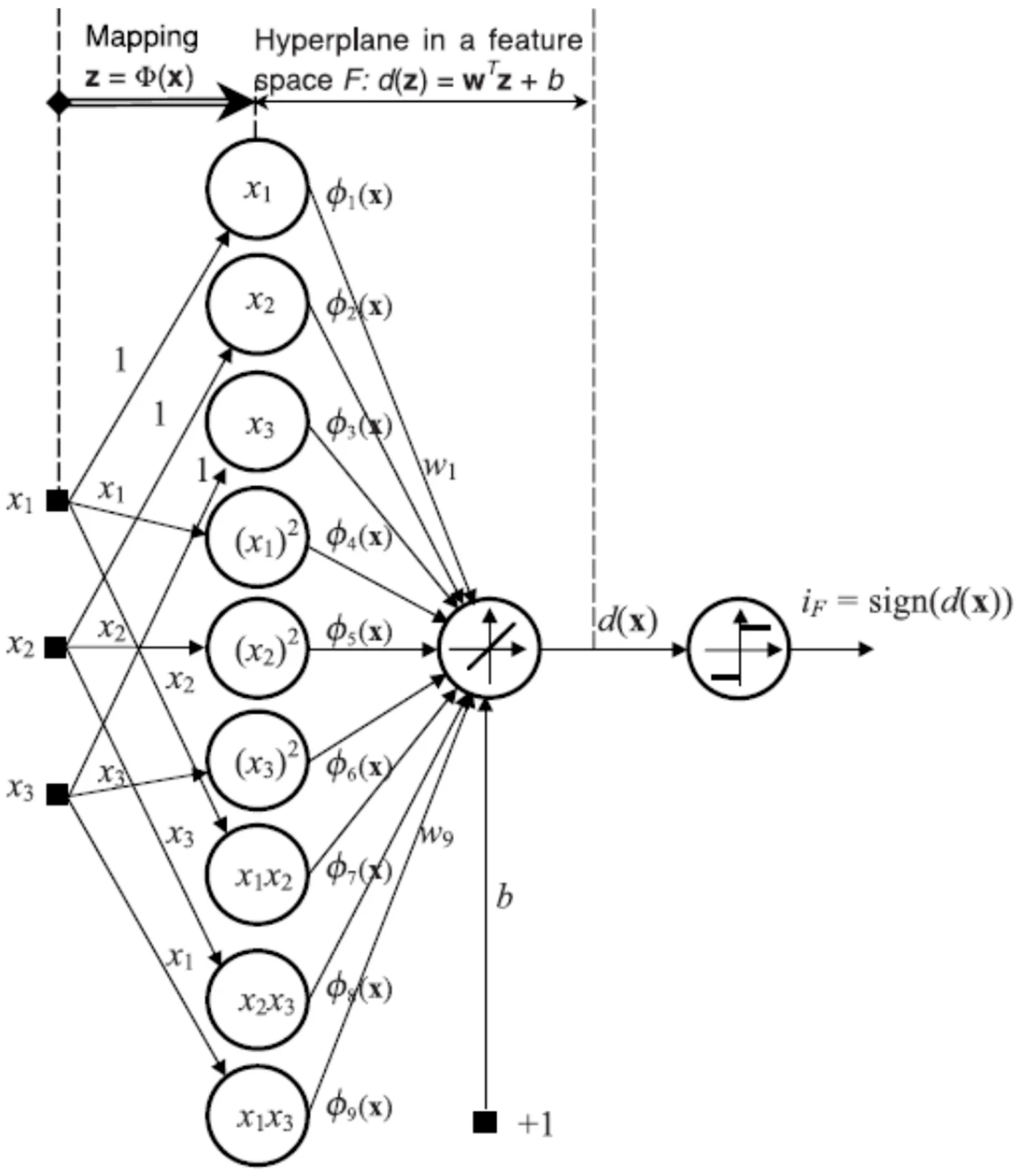
SVM architecture.

Support vector machine (SVM) has demonstrated very good results in solving many different regression or classification problems in various research areas in the last decades. Because of its good generalization capabilities, it became a standard in machine learning methods. Its architecture is similar to the neural network architecture in Figure 5 [36], but the learning algorithm is different. SVMs, like NNs, can approximate every multidimensional non-linear function very accurately.

The main characteristics of the SVM learning algorithm are that it uses so-called kernel functions to map the input data into a multidimensional, so-called feature space, with some mapping function Ф, thereby transforming a non-linear problem into a linear one. In the new feature space, the problem is solved as a linear regression problem.

The input data set *S* contains *m* data pairs, out of which SVM will learn the relationship between input predictors *x_i_* and the target values *y_i_*: *S* = {(*x_i_, y_i_*), *i* = 1, … *m*}. If there are *n* predictors for the target variable, then *x_i_* are *n* dimensional vectors. In the process of training, the components of the weight vector *w* will be obtained. After the process of training, the SVM model will find the *n*-dimensional non-linear approximation function *f*(*x*, *w*), which, of course, will have some deviation ε from the actual target values, which is called the error of the approximation. In the linear regression problem, the approximation function that should be obtained is linear (Equation (4)):

(4) $$f\left(x,w\right)=w^{T}x+a$$

This required function will be obtained by minimizing the term given in Equation (5) [41]:

(5) $$R=\frac{1}{2}{\left\|w\right\|}^{2}+C\left(\sum_{i=1}^{m}\left|y_{i}-f(x_{i},w)\right|\right)$$

where $\left\|w\right\|$ is the norm of the vector of weights *w*, i.e., its length, and *C* is a regularization parameter used in the optimization process of SVM.

SVM has been used for solving many complex problems in hydrology, ecology modelling complex natural processes, text analysis, computer vision and image processing, finance and risk analysis, problems of classification, and many others.

Linear regression (LR) was additionally implemented as a conventional baseline model for comparison with GRNN, RBFNN, and SVM. The LR model applies the linear form given in Equation (4) directly to the original predictors.

## 3. Results and Discussion

### 3.1. Results for Mortars

**Figure 6:**
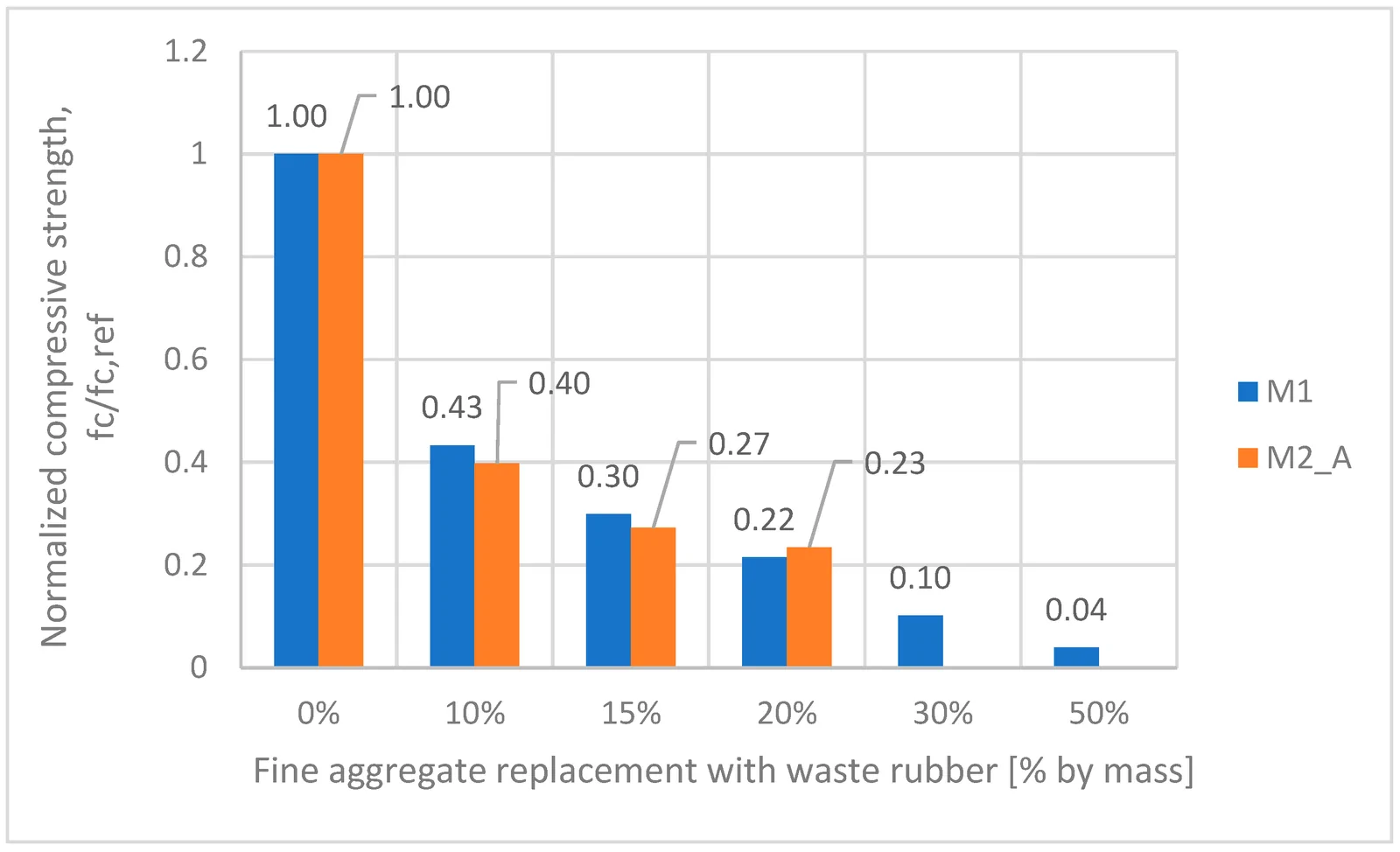
Normalized compressive strength of mortars without (M1) and with admixture (M2_A) at different waste-rubber replacement levels.

The compressive strength of tested mortar samples was calculated as the mean value of at least three tested samples, following the guidelines in MKS EN 1015-11 [32]. The measured compressive-strength values are presented in Table 5, while Figure 6 presents the values normalized with respect to the corresponding reference mortar (0%).

The addition of rubber particles, especially in larger quantities, reduces the strength properties of the mortar. This is because rubber has lower strength compared to natural aggregate, resulting in a weaker bond between the particles and the cement. Consequently, the rubber particles create micro-cracks and increased porosity, leading to reduced mechanical strength.

Mortar containing a superplasticizer admixture exhibits higher strength values, as the chemical admixtures within it allow for a reduction in water demand. The compressive strength of the reference mix with the superplasticizer M2_A is 74.87% higher than that of the reference mix without the admixture M1; the difference is 60.60% for the sample with 10% aggregate replacement by waste rubber and 59.42% for the 15% rubber sample, while for mortars with 20% replacement, there is a 90.54% difference in compressive strength in favour of the mortar M2_A with the superplasticizer.

For mortars without admixtures (M1), compressive strength—compared to the reference sample—decreased by 56.69% at 10% replacement, 70.11% at 15% replacement, 78.45% at 20% replacement, 89.81% at 30% replacement, and 96.06% at 50% replacement.

For M2_A mortars, however, compressive strength—compared to the reference sample—decreased by 60.22% at 10% replacement, 72.75% at 15% replacement, and 76.51% at 20% replacement.

### 3.2. Results for Concrete

The density of hardened concrete was obtained according to [42]. Compressive strength was tested in accordance with MKS EN 12390-3 [33]. A testing machine compliant with MKS EN 12390-4 [34] was used for this purpose.

**Figure 7:**
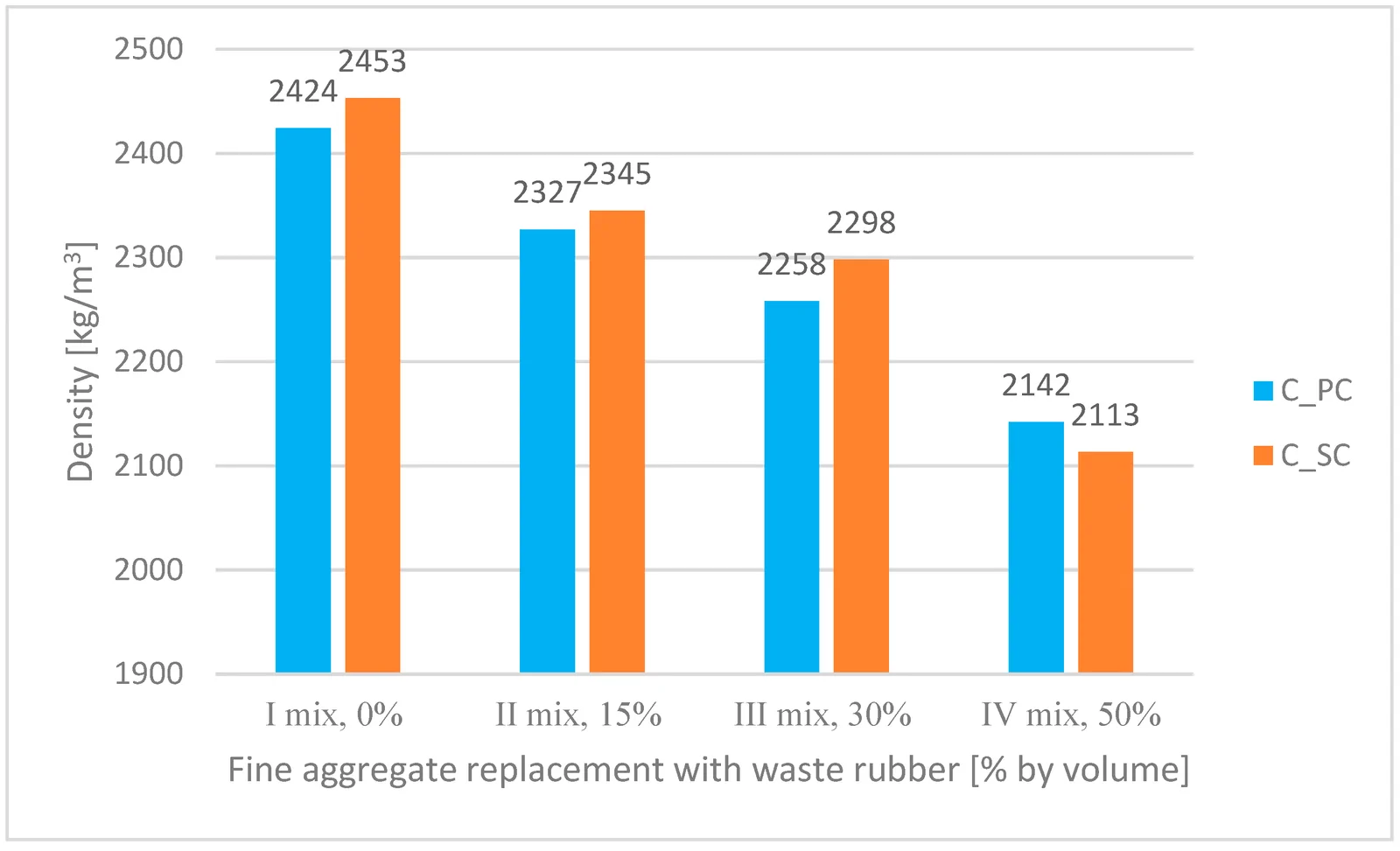
Average density of hardened concrete as a function of percentage share.

The Average density of hardened concrete as a function of percentage share is shown in Figure 7. Due to the low density of the rubber, increasing its percentage content results in concrete that is lighter than the reference mix. For concretes made with ordinary cement (C_PC), a 4% reduction in density is observed with the addition of 15% rubber compared to the reference mix; a 6.8% reduction is noted with 30% rubber, while an 11.6% reduction is observed with the addition of 50% rubber.

For concretes made with sulphate-resistant cement (C_SC), a 4.4% reduction in bulk density is observed with the addition of 15% rubber compared to the control mix; a 6.3% reduction is observed with the addition of 30% rubber, while a 13.9% reduction in bulk density is observed with the addition of 50% rubber compared to the control mix.

**Figure 8:**
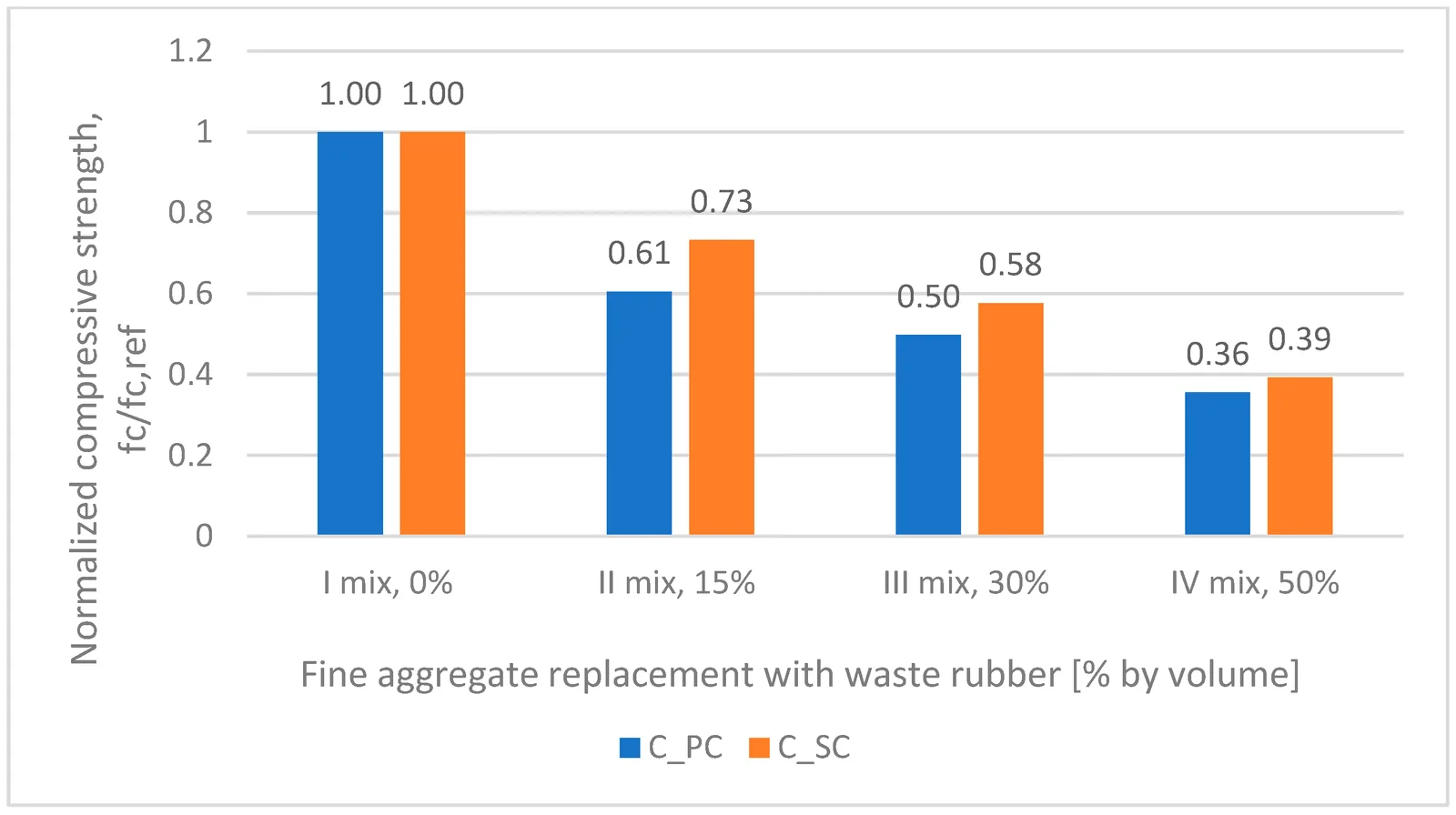
Normalized compressive strength of concrete with Portland cement (C_PC) and sulphate-resistant cement (C_SC) at different waste-rubber replacement levels by volume.

The compressive strength of the tested concrete samples, based on two replicate measurements for each mixture, decreases with increasing waste-rubber content, as shown by the absolute strength values in Table 6 and the normalized values in Figure 8.

Compared to the control mix, the compressive strength of concrete made with ordinary cement (C_PC) decreased by 39.4% at 15% replacement, by 50.2% at 30% replacement, and by 64.4% at 50% replacement.

Compared to the control mix, the compressive strength of concrete made with sulphate-resistant cement (C_SC) decreased by 26.7% at 15% replacement, by 42.3% at 30% replacement, and by 60.7% at 50% replacement.

For example, with a 15% replacement of aggregate with waste rubber and using ordinary cement, the 28-day compressive strength is 34.85 MPa. With the same percentage of rubber and sulphate-resistant cement, the 28-day compressive strength is 47.65 MPa. The use of concrete incorporating waste tyres offers additional benefits, such as reduced CO_2_ emissions, resource and energy savings, and lighter structures.

**Figure 9:**
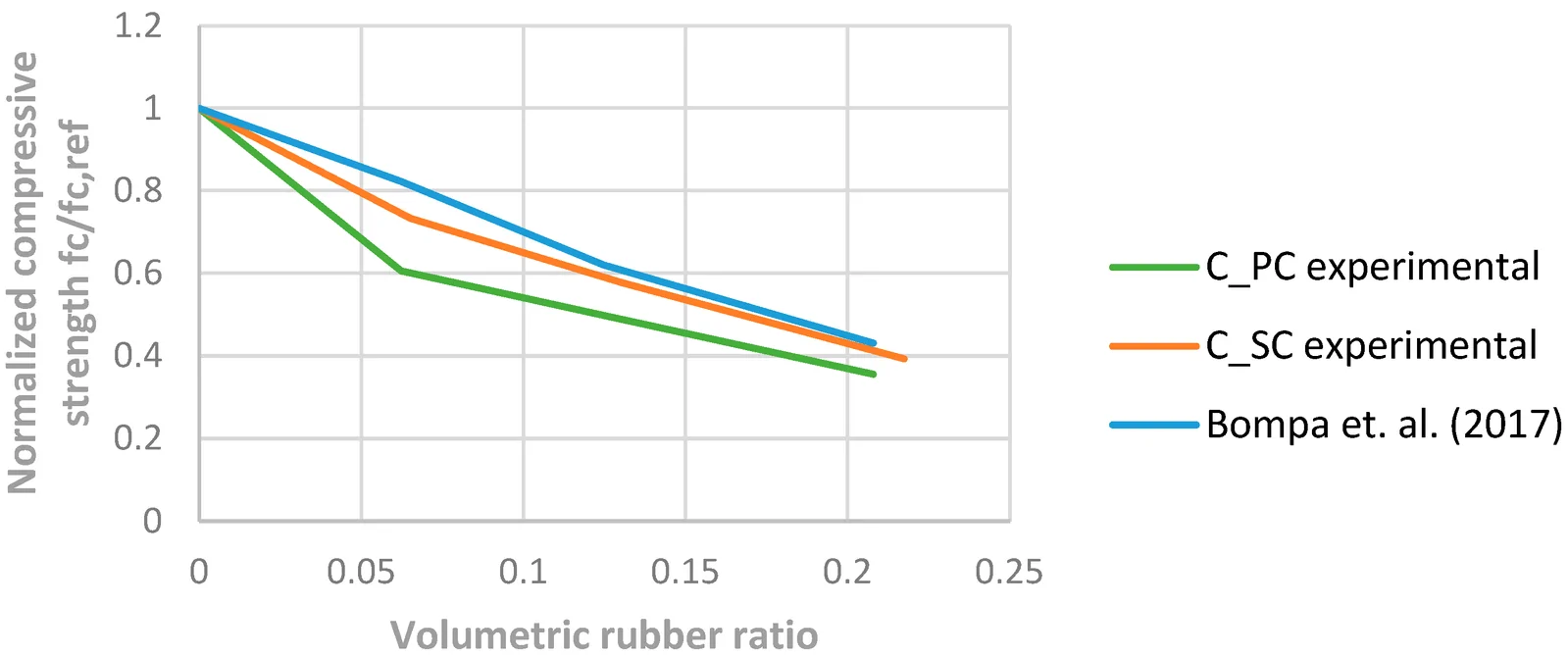
Comparison of the experimental normalized compressive strength of C_PC and C_SC with the predictive relationship proposed by Bompa et al. [43] for fine-aggregate replacement (λ = 2.43).

To benchmark the experimentally observed strength-degradation trend against an independent model, the normalized compressive-strength results were compared with the predictive relationship proposed by Bompa et al. [43] Figure 9. In the model of Bompa et al., the volumetric rubber ratio is defined relative to the total mineral-aggregate volume. Since the replaced aggregate was the fine 0/4 mm fraction, λ = 2.43 was adopted.

The comparison shows that both experimental series follow the general strength-degradation trend predicted by Bompa et al. The C_SC results show closer agreement with the predictive curve, particularly at higher rubber ratios, whereas C_PC exhibits a more pronounced strength reduction.

### 3.3. Predictive Models for the Strength of Mortars

The dataset for the mortars has 40 rows and 5 columns (for the target and 4 predictors). Each row represents an individual experimental result. The predictors are: rubber percentage (by mass), admixture (this predictor has textual values; some of the mortars have admixture, and some of them do not have), w/c (water-cement ratio), and density (kg/m^3^), and the target is the strength of the mortar (in MPa). The admixture predictor was treated in DTREG as a categorical (text) variable. For mixtures containing the superplasticizer, SUPERFLUID 21M1M (Ading) was entered as the categorical value, whereas the corresponding field was left blank for mixtures without a superplasticizer. All 40 mortars have the same type of cement and the same type of aggregate. The complete mortar dataset used for model development and validation is provided in Supplementary Data S1.

For predicting the strength of the mortars, the predictive models that can work efficiently with small datasets were used: the SVM model, GRNN, and RBF NN, within the DTREG software. The three ML (machine learning) models were built with the original dataset and also with a normalized dataset. The normalization was done with the *Ln* function in order to obtain more homogeneous values of the variables, which improved the accuracy of the models significantly. The LR (linear regression) model was also built as a conventional regression model for comparison with the ML models (on the original and normalized dataset).

The accuracy of the models is estimated with the standard estimators: (1) R^2^—coefficient of determination, which measures the general goodness of fit of the model (i.e., how well the approximation function fits the data points, or how much is the predictive performance of the model); (2) r—the coefficient of correlation between actual and predicted target values (it reflects the degree of linear association between them); and (3) MAPE—mean absolute percentage error, which measures the magnitude of the prediction error, expressed as a percentage.

The method for testing the models is Leave-One-Mixture-Out validation. This validation method is a cross-validation technique used when the dataset has several rows with identical values for the predictors. In the dataset for mortars, there are 10 mixtures in the 40 rows. With this method, the data is grouped into distinct mixtures. All 4 rows that belong to one mixture are held out as the test set, and the model is trained on the remaining mixtures. This process is repeated until every mixture has served as the test set once. It measures how well the model generalizes to completely new unseen mixtures, preventing data leakage between the training and test sets.

The results for the accuracy of the 4 models for the test data, using the original input data set without normalization, are given in Table 7.

**Figure 10:**
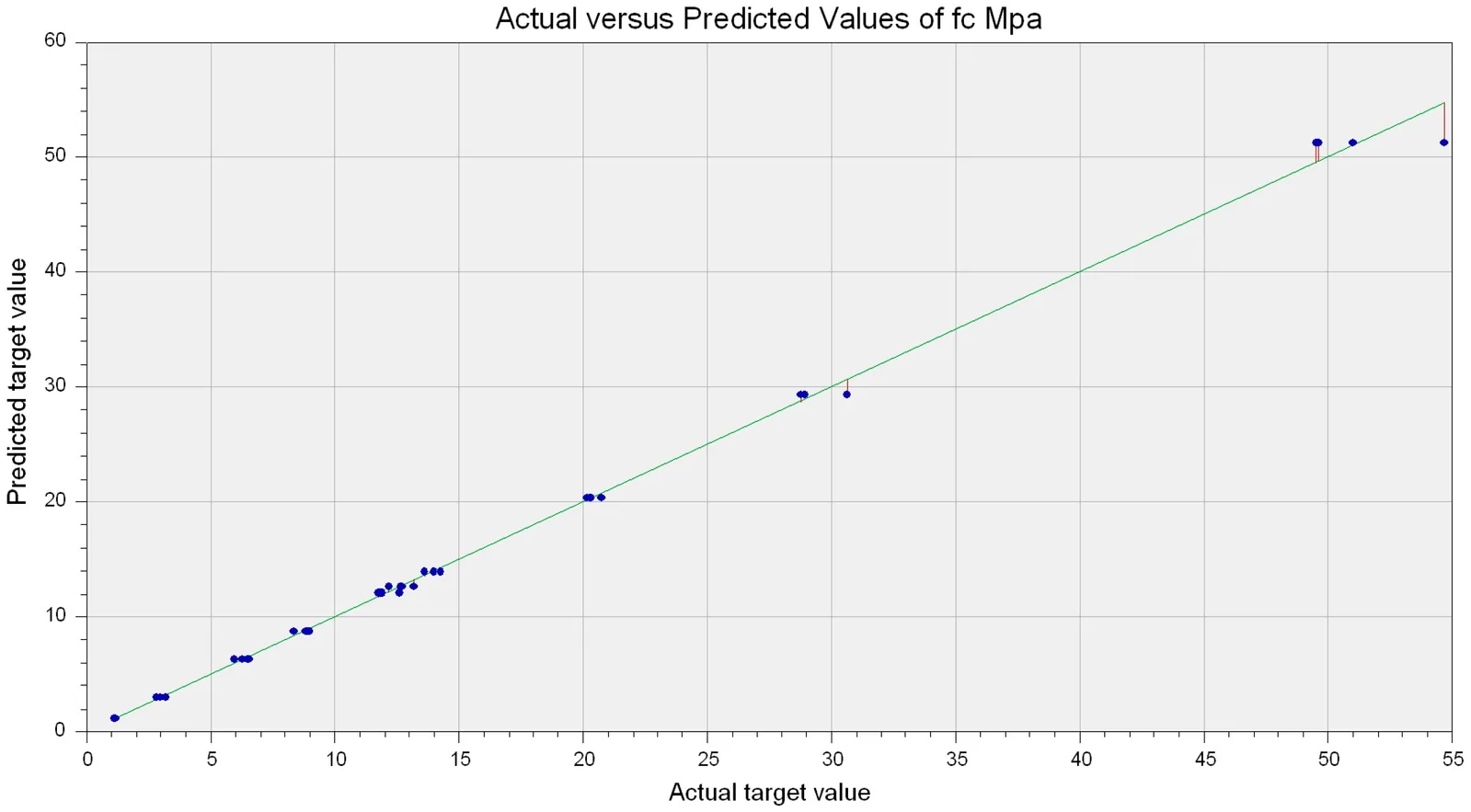
Actual versus predicted values of the target: strength of the mortars (GRNN model on original dataset using Leave-One-Mixture-Out validation).

GRNN model demonstrated excellent predictive abilities, although there were only 10 different mixtures in this dataset of 40 rows. SVM and RBF NN without normalization exhibited poor predictive performance, which is expected for distance-based algorithms, because they are highly sensitive to unscaled variable magnitudes when trained on very small datasets (10 distinct mixtures in 40 rows). Figure 10 shows the chart of predicted and actual values of the target for the GRNN as the model with the best performance. The LR model demonstrated the poorest overall performance because the relationship between variables is highly non-linear, and the LR model fails to capture it.

The ranking of the importance of predictors for the GRNN model is the following: (1) rubber percentage (maximum importance), (2) density, (3) w/c ratio, and (4) admixture. Physically, rubber content has the greatest influence because replacing mineral aggregate with the softer rubber phase reduces strength. Density reflects changes in composition and compactness, while the w/c ratio affects matrix porosity and strength. Since rubber content and density are strongly correlated, the predictor-importance ranking should be interpreted with caution and should not be regarded as indicating independent causal effects of the individual predictors.

Figure 10 illustrates the relationship between the actual and predicted values of the target for the GRNN model.

In DTREG software, the hyper-parameters of the models (like RBF NN, SVM, and GRNN) are automatically optimized by the software’s built-in algorithms, but the software also allows users to manually adjust these parameters to better match the specific structure and characteristics of their dataset. For the models on the original dataset, some of the adjusted hyperparameters were: (1) for the GRNN, the most important parameter sigma was adjusted to 0.0001 (min) and 5 (max), type of kernel function: Gaussian. (2) For RBF NN: number of neurons was adjusted to 10. (3) For SVM: kernel function is chosen as RBF, parameter C = 128.5.

Because of these poor results for SVM and RBF, the numeric variables were normalized with the *Ln* function in order to obtain more homogeneous data, which drastically improved the accuracy of all models. The results for the accuracy of the models for the test data with normalized data are presented in Table 8.

Both GRNN and SVM achieved excellent accuracy, while RBF had a slightly higher MAPE, despite the high correlation for r = 0.986 and high goodness of fit for R^2^ = 97.12%. Again, the LR model achieved the lowest performance. The linear model successfully captures the global monotonic trend, resulting in high R^2^ and r, but it fails to fit the local nonlinear curvature. The discrepancy between R^2^ and MAPE is critical and stresses the necessity of non-linear ML models to adequately capture both local curvature and global behaviour.

Some of the hyperparameters that were adjusted for the 3 ML models on the normalized dataset were: (1) for GRNN: min sigma = 0.0001, max sigma = 3, kernel function: Gaussian, (2) for RBF NN: max neurons:15, (3) for SVM: kernel function: RBF, C = 1088.9.

### 3.4. Predictive Models for Compressive Strength of Concrete

The dataset for the concrete has 48 rows with 5 columns (for values of the target value and 4 predictors). Each row represents an individual experimental result. The target variable is the strength of the concrete (in MPa), and the predictors are rubber percentage (by volume), cement type, age (in days), and density (kg/m^3^). The complete concrete dataset is provided in Supplementary Data S1.

Three ML predictive models were developed using the original dataset within the DTREG software, using also the SVM model and the two neural networks: GRNN and RBF NN. The LR model was also built for comparison with the three ML models. The validation method for all models was Leave-One-Mixture-Out. This dataset of 48 rows has 24 mixtures, and because of the larger dataset and greater number of mixtures, the results on the original dataset were significantly improved. The estimators of the accuracy of the models are the same as for the models for predicting the strength of mortars.

The results for the accuracy of the 4th models on the original dataset for the test data are presented in Table 9.

**Figure 11:**
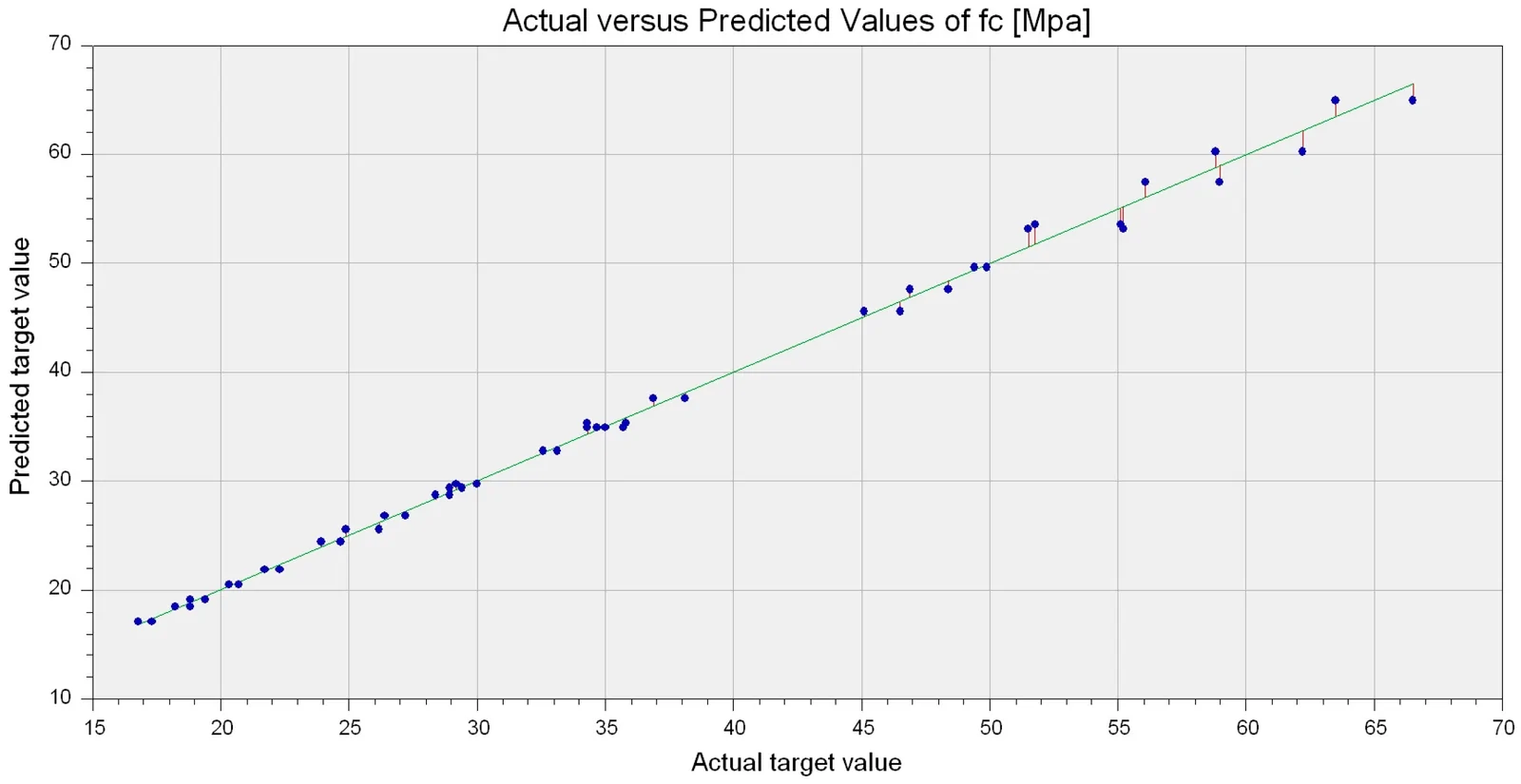
Actual versus predicted values of the target: strength of the concrete (GRNN model on original dataset using Leave-One-Mixture-Out validation).

The results for all three ML models for the test data are excellent, which means that all three ML models demonstrated excellent predictive abilities, and because all non-linear ML models achieved exceptional predictive precision on the raw data, further scaling for the 24-mixture concrete dataset was not necessary. The relatively low MAPE error and strong linear correlation observed for the LR model on raw concrete data indicate that the underlying relationships between the input variables possess a monotonic character, but the results also indicate that non-linear machine learning algorithms remain essential in capturing the remaining complex non-linear interactions between the target and predictors. The results confirm that, on the unscaled concrete dataset, all 3 ML models outperformed the LR model, and the GRNN demonstrated the best predictive performance. Figure 11 shows the actual versus predicted target values.

For comparison, Ref. [22] reported a test-set MAPE of 21.64%, whereas the best-performing concrete model in the present study, GRNN, achieved a substantially lower MAPE of 3.8% on the original, non-normalized MPa scale. The lower MAPE may be associated with the more homogeneous experimental dataset and differences in the modelling approaches, with GRNN used in the present study and a multilayer feed-forward ANN used in Ref. [22].

Some of the hyperparameters adjusted in the three ML models developed on the original dataset for concretes are: (1) for GRNN: min sigma = 0.0001, max sigma = 5, kernel function: Gaussian, (2) for RBF NN: maximal neurons = 100, (3) for SVM: kernel function is RBF and C is 20,029.5.

The DTREG software also gives an estimation of the importance of the predictors. For ranking of the predictors for the GRNN, it is as follows: (1) rubber percentage (with maximum importance), (2) cement type, (3) age (in days), (4) density. Physically, rubber content directly affects the proportion of the softer rubber phase, while cement type and age influence the development and properties of the cementitious matrix. Density reflects changes in composition and compactness. Since rubber content and density are strongly correlated, the predictor-importance ranking should be interpreted with caution and should not be regarded as indicating independent causal effects of the individual predictors.

The low prediction errors obtained for the three ML models indicate good predictive performance within the investigated experimental domain and show that the nonlinear relationships between the predictors and the target were successfully captured by the models.

The LR model presented the lowest predictive accuracy among all evaluated algorithms (models), which is attributed to the presence of significant complex nonlinear interactions between predictors and target, which can not be adequately modelled with a linear framework.

A critical consideration in material property prediction is ensuring reliable model generalization despite a small dataset (40 rows with 10 mixtures for mortars and 48 rows with 24 mixtures for concretes). The Leave-One–Mixture-Out (LOMO) validation used in this research addresses this directly by isolating one mixture during testing; also, using ML architectures that are specifically constructed and theoretically optimized for small datasets, and the low prediction errors on test data achieved under LOMO validation indicate good predictive performance for unseen mixtures within the investigated dataset without overfitting.

It is important to stress that all high values for R^2^ > 0.98 are for the testing data and report out-of-sample performance evaluated strictly on unseen test mixtures during LOMO validation without leaking information between training and testing data. Achieving high precision on unseen mixtures indicates that the models captured nonlinear relationships within the investigated dataset and reduces concerns regarding memorizing training noise (overfitting). These high values of R^2^ may also be related to the high-quality dataset, which was derived strictly from controlled laboratory formulations with standardized testing procedures and the presence of replicate measurements, which ensured high-quality, clean data with minimal noise, allowing the ML models to capture the relationships within the investigated dataset accurately.

### 3.5. Significance of This Study

The main contribution of this study is the new experimental dataset and the development of predictive models for estimating compressive strength within the investigated mixture space. Owing to the limited range and sources of the constituent materials, the developed models should primarily be regarded as interpolation tools for mixtures with characteristics similar to those investigated in this study. They can support preliminary strength estimation and mixture design when the required predictors are known but are not intended to replace laboratory testing.

A significant aspect of the result dataset is that, despite the reduced strength resulting from the use of rubber in the concrete, concrete with a high content of fine rubber particles could potentially be suitable for non-structural applications. The limitations regarding the use of concrete with a high content of rubber particles in non-structural elements could be addressed by adding other materials, such as steel fibers [44] to increase the compressive strength; production of special types of concrete such as self-compacting and rolled compacted concrete [45,46]; lightweight geopolymer concrete with incorporated pumice and rubber [47]. Further validation using mixtures produced with different constituent materials and material sources will be required before broader application.

Future work could extend the training space beyond the experimentally tested mixtures by incorporating additional cases and rubber-content combinations generated using validated numerical mesoscale models, such as the model developed by Jurado et al. [48].

## 4. Conclusions

The aim of this study was to test the strength of mortars and concrete prepared with varying content of waste tyre rubber, as well as to develop models for predicting the strength of such mortar/concrete.

Standard compressive strength testing was carried out on 48 concrete and 40 mortar specimens prepared with varying rubber content: 10%, 15%, 20%, 30%, and 50%.

The empirical results from these tests showed that increasing the percentage of waste rubber in the mixture designs negatively affects compressive strength, reducing it up to 96.06% for mortars, up to 64.4% for concrete made with ordinary Portland cement, and up to 60.7% for concrete made with sulphate-resistant cement.

The results from the compressive strength testing were used to develop strength predictive models. Three soft computing techniques were implemented using the DTREG predictive modelling software: the General Regression Neural Network (GRNN), Radial Basis Function Neural Network (RBFNN) and Support Vector Machine (SVM). The conventional linear regression model (LR) was also implemented for the purpose of comparison with the 3 ML models. They were trained using the experimentally measured results for the various mixtures.

The GRNN model demonstrated excellent predictive abilities, outperforming the predictive abilities of the other models for both the strength of mortars and concretes. In all models, the rubber percentage was shown to be the main predictor of the compressive strength.

The results also showed that, despite the reduced strength resulting from the use of rubber in the concrete, concrete with a high content of fine rubber particles could be suitable for non-structural applications.

## Figures and Tables

**Table 1:** Mix proportions of Mortar M1 with different waste-rubber replacement levels by mass.

	M1_0%	M1_10%	M1_15%	M1_20%	M1_30%	M1_50%
Cement Type	CEM I	CEM I	CEM I	CEM I	CEM I	CEM I
Cement [g]	450	450	450	450	450	450
w/c	0.7	0.7	0.7	0.7	0.9	1.1
Aggregate Type	CEN standard sand	CEN standard sand	CEN standard sand	CEN standard sand	CEN standard sand	CEN standard sand
Fine aggregate [g]	1350	1215	1147.5	1080	945	675
Waste Rubber [g]	0	135	202.5	270	405	675

**Table 2:** Mix proportions of Mortar M2_A with different waste-rubber replacement levels by mass.

	M2_0%	M2_10%	M2_15%	M2_20%
Cement Type	CEM I	CEM I	CEM I	CEM I
Cement [g]	450	450	450	450
w/c	0.45	0.45	0.45	0.45
Aggregate Type	CEN standard sand	CEN standard sand	CEN standard sand	CEN standard sand
Fine aggregate [g]	1350	1215	1147.5	1080
Waste Rubber [g]	0	135	202.5	270
Admixture Type	Superfluid 21M1M	Superfluid 21M1M	Superfluid 21M1M	Superfluid 21M1M
Admixture [g]	2.25	2.93	3.6	5.85

**Table 3:** Mix proportions of concrete C_PC with different waste-rubber replacement levels by volume, expressed per 1 m^3^ of concrete.

Mix	0/4 mm (kg)	4/8 mm (kg)	8/16 mm (kg)	16/32 mm (kg)	Cement (kg)	Water (kg)	Admixture (kg)	Rubber (kg)
0%	740	220	480	340	380	190	2.5	0
15%	628.8	220	480	340	380	190	2.5	27.5
30%	517.8	220	480	340	380	190	2.5	55.2
50%	369.8	220	480	340	380	190	2.5	92.0

**Table 4:** Mix proportions of concrete C_SC with different waste-rubber replacement levels by volume, expressed per 1 m^3^ of concrete.

Mix	0/4 mm (kg)	4/8 mm (kg)	8/16 mm (kg)	16/32 mm (kg)	Cement (kg)	Water (kg)	Admixture (kg)	Rubber (kg)
0%	800	180	490	370	360	180	2.3	0
15%	679.8	180	490	370	360	180	2.3	29.8
30%	559.8	180	490	370	360	180	2.3	59.7
50%	399.9	180	490	370	360	180	2.3	99.3

**Table 5:** Compressive strength of two types of mortars with different percentages of waste rubber.

**Mortar M1**	**M1_0%**	**M1_10%**	**M1_15%**	**M1_20%**	**M1_30%**	**M1_50%**
*fc* [Mpa]	30.65	12.73	8.34	5.96	2.97	1.14
	28.78	13.17	8.86	6.26	3.2	1.12
	28.8	12.68	8.81	6.54	2.81	1.17
	28.92	12.16	9.01	6.5	2.96	1.19
*fc*, _average_ [Mpa]	29.29	12.69	8.76	6.32	2.99	1.16
±SD	0.910	0.413	0.289	0.267	0.161	0.031
**Mortar M2_A**	**M2_0%**	**M2_10%**	**M2_15%**	**M2_20%**		
fc [Mpa]	49.54	20.17	14.24	11.94		
	54.7	20.27	13.98	11.75		
	51.01	20.33	13.99	12.62		
	49.61	20.72	13.62	11.82		
*fc*, _average_ [Mpa]	51.22	20.37	13.96	12.03		
±SD	2.42	0.24	0.26	0.40		

**Table 6:** Compressive strength of concrete mixtures at 28 days with different waste-rubber replacement levels by volume.

Concrete Type	Rubber Replacement [% by Volume]	f_c,1_ [MPa]	f_c,2_ [MPa]	Mean [MPa]	SD [MPa]
C_PC	0%	56.1	59.0	57.55	2.05
C_PC	15%	34.7	35.0	34.85	0.21
C_PC	30%	28.4	28.9	28.65	0.35
C_PC	50%	20.7	20.3	20.50	0.28
C_SC	0%	66.5	63.5	65.00	2.12
C_SC	15%	46.9	48.4	47.65	1.06
C_SC	30%	36.9	38.1	37.50	0.85
C_SC	50%	24.9	26.2	25.55	0.92

**Table 7:** The accuracy of the predictive models for the test data for predicting the strength of mortars using original dataset.

	R^2^ (%)	r	MAPE (%)
GRNN	99.27	0.996	4.6
RBF NN	40.24	0.729	52.8
SVM	78.1	0.922	21.76
LR	28.9	0.745	151.4

**Table 8:** The accuracy of the predictive models for the test data for predicting the strength of mortars using normalized dataset.

	R^2^ (%)	r	MAPE (%)
GRNN	99.65	0.998	3.9
RBF NN	97.12	0.986	16.4
SVM	98.69	0.994	6.34
LR	93.80	0.978	51.8

**Table 9:** The accuracy of the predictive models for the test data for prediction of strength of concrete on original dataset.

	R^2^ (%)	r	MAPE (%)
GRNN	98.4	0.992	3.8
RBF NN	97.6	0.988	4.8
SVM	97.99	0.989	5.3
LR	87.5	0.936	13.5

## Data Availability

The original contributions presented in this study are included in the article/Supplementary Material. Further inquiries can be directed to the corresponding author.

## Supplementary Materials

The following supporting information can be downloaded at https://www.mdpi.com/article/10.3390/ma19183993/s1, Figure S1: Particle-size distribution of the waste rubber; Data S1: Experimental datasets used for the development and validation of the mortar and concrete predictive models.
