# Supplementary material for: Empirical Determination and Modelling of Compressive Strength of Cement Composites with Waste Tyre Rubber
Source: Materials (Basel). 2026 Sep 19;19(18):3993. doi: 10.3390/ma19183993 (PMC13608672; doi:10.3390/ma19183993)
Supplement: Supplementary file 1 [file materials-19-03993-s001.zip › materials-4557288-Supplementary Materials.pdf]

15.9%

Дијаметар / Diameter [mm]	Процент / Percentage [%]
63	100.0
31.5	100.0
20	100.0
16	100.0
8	99.6
6	97.9
4	96.2

84.1%

Дијаметар / Diameter [mm]	Процент / Percentage [%]
2	84.1
1	45.1
0.6	14.0
0.5	6.3
0.25	0.6
0.2	0.4
0.125	0.1

0.0%

Дијаметар / Diameter [mm]	Процент / Percentage [%]
0.063	0.0

0.0%

Дијаметар / Diameter [mm]	Процент / Percentage [%]

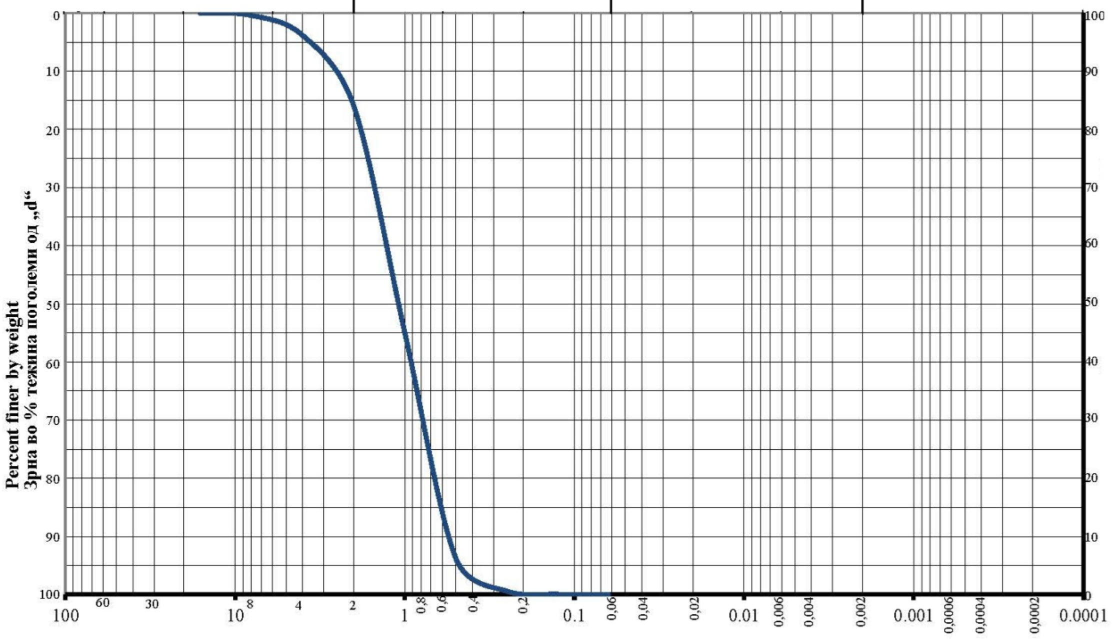

Figure S1. Particle-size distribution of the waste rubber.
